# Spatiotemporal Characteristics of Bacterial Communities in Estuarine Mangrove Sediments in Zhejiang Province, China

**DOI:** 10.3390/microorganisms13040859

**Published:** 2025-04-09

**Authors:** Liqin Yao, Maoqiu He, Shoudian Jiang, Xiangfu Li, Bonian Shui

**Affiliations:** 1 Fisheries College, Zhejiang Ocean University, Zhoushan 316022, China; yaoliqin@zjou.edu.cn (L.Y.); jiangshoudian@zjou.edu.cn (S.J.); 2 Fujian Key Laboratory of Marine Carbon Sequestration, Xiamen University, Xiamen 361000, China; 3 State Key Laboratory of Tropical Oceanography, South China Sea Institute of Oceanology, Chinese Academy of Sciences, Guangzhou 510260, China; xfli@scsio.ac.cn

**Keywords:** Ao River estuary, artificially introduced mangrove, bacterial diversity, seasonal variation

## Abstract

Mangrove forests are intertidal ecosystems that harbor diverse microbial communities essential for biogeochemical cycles and energy flow. This study investigated the seasonal and spatial patterns of bacterial communities in the artificially introduced mangrove sediments of the Ao River estuary using 16S rRNA gene amplicon high-throughput sequencing. Alpha diversity analyses indicated that the bacterial community diversity in the mangrove sediments of the Ao River estuary was similar to those of natural-formed mangroves, with the Shannon index ranging from 5.16 to 6.54, which was significantly higher in winter compared to other seasons. The dominant bacterial phyla included Proteobacteria (43.65%), Actinobacteria (11.55%), Desulfobacterota (11.16%), and Bacteroidetes (5.52%), while beta diversity analysis revealed substantial differences in bacterial community structure across different seasons and regions. For instance, the relative abundance of Woeseiaceae and Bacteroidota during the summer was significantly higher than that observed in other seasons. And the relative abundance of Bacillaceae in autumn and winter increased by one order of magnitude compared to spring and summer. Woeseiaceae, Desulfobulbaceae, Thermoanaerobaculaceae, and Sva1033 (family of Desulfobacterota) exhibited significantly higher relative abundance in the unvegetated area, whereas Bacillaceae and S085 (family of Chloroflexi) demonstrated greater abundance in the mangrove area. Seasonal variations in bacterial community structure are primarily attributed to changes in environmental factors, including temperature and salinity. Regional differences in bacterial community structure are primarily associated with environmental stressors, such as wave action, fluctuations in salinity, and organic matter content, which are further complicated by seasonal changes. This study is significant for understanding the microbial diversity and seasonal dynamics of estuarine mangrove wetlands, and it contributes to the assessment of mangrove wetland restoration efforts in Zhejiang Province, providing important guidance for the development of strategies to maintain the health of mangrove ecosystems in the future.

## 1. Introduction

Mangrove forests, which are coastal wetland ecosystems dominated by shrubs or trees, cover 60–75% of tropical and subtropical coastlines and provide essential ecological and economic services, including biodiversity support, coastal protection, sediment trapping, water purification, and atmospheric regulation [1,2]. Furthermore, mangroves rank among the most productive ecosystems globally and make a significant contribution to the storage of carbon in coastal and marine ecosystems (“Blue Carbon”) [3,4,5]. Sediment microbial communities are integral to mangrove ecosystems, facilitating organic matter degradation and nutrient cycling processes that are essential for ecosystem function and maintenance [6,7]. Interactions between mangrove plants and sediment microorganisms are closely intertwined. Plant litter, comprising leaves and branches, enriches sediments with significant organic carbon and nutrients, thus supporting microbial metabolism, which is further enhanced by root exudates [8,9]. Conversely, sediment microorganisms facilitate vital biogeochemical processes, including carbohydrate degradation, nitrogen fixation, and sulfate reduction, supplying plants with essential nutrients and transferring carbon and energy throughout the food web via animal consumption [10,11,12]. Additionally, mangrove microorganisms contribute to environmental remediation by degrading pollutants, including polycyclic aromatic hydrocarbons, heavy metals, petroleum, and pesticides [13,14,15,16].

Since the beginning of the 21st century, there has been a growing recognition of the ecological importance of mangroves in coastal zones and their roles in global change, which has spurred increased international efforts to protect and restore these ecosystems [17]. China has implemented extensive ecological restoration projects, including large-scale mangrove planting, leading to significant increases in mangrove areas [18,19]. Successful mangrove restoration encompasses not only area expansion but also alterations in plant–microbe interactions, which significantly influence the degree of restoration [20,21]. Some studies have demonstrated that particular bacterial taxa increase their abundance during the mangrove restoration process [22,23]. For instance, Desulfobacteraceae thrive in the anoxic sediments of rehabilitated mangroves, where they mediate sulfur cycling through dissimilatory sulfate reduction and their elevated abundance correlates with improved organic matter mineralization [22]. Similarly, nitrogen-fixing bacteria like Rhizobiaceae show marked proliferation in the soils of restored mangroves, forming symbiotic relationships with mangrove roots to enhance nitrogen bioavailability [23]. These taxa’s metabolic activities directly support mangrove growth by converting atmospheric N_2_ into bioavailable ammonium while simultaneously improving soil fertility. With the advent of high-throughput sequencing technologies, the genomic analysis of microbial communities has become more accessible, enabling researchers to identify specific genes associated with key metabolic processes, such as carbon cycling, nitrogen fixation, and pollutant degradation [24]. Understanding the genetic functions of microorganisms in mangrove ecosystems provides a more detailed insight into their ecological roles, including their contributions to nutrient cycling and ecosystem health [25].

Current restoration assessments primarily focus on metrics such as forest area, vegetation structure, productivity, and biodiversity [26]. The critical role of belowground processes, particularly the contribution of soil microorganisms to litter decomposition and soil nutrient mineralization, is often overlooked, despite evidence indicating that bacterial community structure and diversity can serve as indicators of ecological change [18,27].

The Ao River estuary, located at the northern edge of Chinese mangrove distribution in Zhejiang Province, sustains a stable population of the artificially introduced mangrove species *Kandelia obovata* [28]. This region is characterized by a subtropical marine monsoon climate, marked by warm, humid conditions and abundant rainfall, with an average annual temperature of 16.8 °C [29]. Ongoing government-led restoration efforts have led to the continuous expansion of the Ao River mangrove area, thereby demonstrating the effectiveness of these conservation initiatives [30]. However, research on microbial communities in artificially planted mangrove areas within Zhejiang Province remains limited, especially concerning assessments of mangrove ecological restoration from a microbial perspective. Therefore, this study aimed to characterize the bacterial community structure in Ao River estuary mangrove sediments across different seasons and regions through high-throughput sequencing analysis, and to investigate the relationships between bacterial communities and environmental factors. This research will contribute to a more comprehensive understanding of estuarine mangrove ecosystems and provide insights into the effectiveness of mangrove wetland restoration efforts in Zhejiang Province.

## 2. Materials and Methods

### 2.1. Study Area and Sample Collection

**Figure 1:**
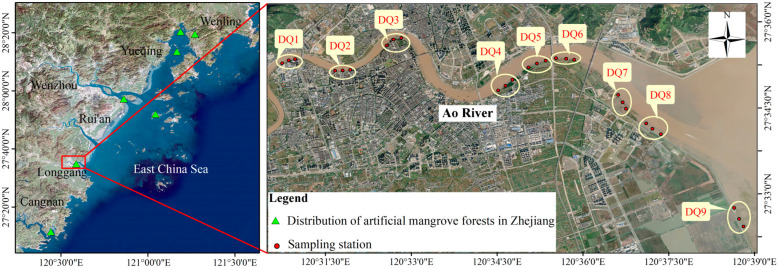
Satellite map of sampling sites in the Ao River estuary mangroves.

Nine sampling sections (DQ1–DQ9) were established along the Ao River estuary mangroves, extending from upstream to the estuary, with three sampling sites per section (M1–M3) (Figure 1). Among these sections, DQ5 and DQ6 represented mature mangrove forests (>10 years old), while DQ1–DQ4 and DQ7–DQ8 comprised younger forests. DQ9 served as an unvegetated control. Sediment samples were collected in December 2021 (winter), May 2022 (spring), August 2022 (summer), and November 2022 (autumn), with a total of 107 samples collected (Table S3). At each site, 30 mL of surface sediment was collected in duplicate using a sterilized syringe (with the tip removed), which was inserted vertically into the sediment to a depth of 10 cm. Samples were stored in sterile centrifuge tubes and subsequently frozen for transport. A portion of each sample was stored at −80 °C for microbial analysis, while the remainder was freeze-dried and subsampled for soil particle size analysis, as well as the determination of total carbon (TC) and total nitrogen (TN) content.

### 2.2. Determination of Environmental Parameters

The pH, temperature, and salinity of interstitial water were measured in situ using a portable water quality analyzer (YSI 2000). Sediment particle size distribution was determined utilizing a laser particle size analyzer (Bettersize 2600, Better Sans Instruments Co., Ltd., Jinan, China). Clay, silt, and sand content were analyzed by dispersing 2 g of dried sediment in 3 mL of 0.5 mol/L sodium hexametaphosphate solution. After a 2 min shake, an 8 h rest, and a further shake, 4–5 drops of the mixture were placed into the laser particle size analyzer [31]. Water-soluble salt content was measured using a mass method with a soil–water ratio of 5:1. Briefly, 50 g of sieved sediment was mixed with 250 mL of ultrapure water in a 500 mL drying Erlenmeyer flask. After a 3 min shake, the mixture was filtered, and 50 mL of the filtrate was evaporated to dryness in an oven [32]. Total carbon (TC) and total nitrogen (TN) content were analyzed using an elemental analyzer (Thermo Fisher Scientific FLASH 2000, Thermo Fisher Scientific, Waltham, MA, USA).

### 2.3. DNA Extraction, PCR Amplification, and Sequencing

Microbial DNA was extracted from sediment samples using DNeasy PowerSoil Pro kit (Qiagen, Hilden, Germany) according to the manufacturer’s protocol. DNA integrity and quality were assessed using 1% agarose gel electrophoresis and a NanoDropOne micro-volume spectrophotometer (Thermo Fisher Scientific, Waltham, MA, USA). The extracted DNA served as template for PCR amplification of bacterial 16S rRNA gene V3-V4 region using primers 341F (5′-CCTAYGGGRBGCASCAG-3′) and 806R (5′-GGACTACNNGGGTATCTAAT-3′) [33]. Purified PCR products were utilized to construct sequencing libraries, which were sequenced using the Illumina NovaSeq 6000 platform (PE250). The sequence data are available in the National Center for Biotechnology Information (NCBI) database under accession number PRJNA1133737.

### 2.4. Data Analysis

Raw sequences were processed using DaDa2 pipeline in QIIME2 (v2023.5) to obtain high-quality merged sequences [34]. These sequences were clustered into operational taxonomic units (OTUs) at a 97% similarity threshold using the vsearch cluster-features-de-novo algorithm. OTU annotation was performed against the SILVA (v138) and Greengenes (v13.8) databases using the qiime feature-classifier algorithm [35]. Microbial alpha diversity was characterized using the Shannon index, which measures community diversity based on species richness and evenness, calculated using the following formula:

*H*′ = −∑ (*P_i_* · ln (*P_i_*)) (1)

where *H*′ is the Shannon diversity index and *P_i_* is the proportion of each species in the sample [36]. Beta diversity analysis was visualized using Bray-Curtis distance matrices, and in this study, non-metric multidimensional scaling (NMDS) and clustering were employed for representation. Alpha and beta diversity analyses, correlation analysis, and redundancy analysis (RDA) were conducted in R (v4.3.0) software, with the pheatmap and vegan packages used for clustering and generating heatmaps of bacterial genera. Microbial community functional prediction was accomplished using PICRUSt2 (v2.4.2) software. The linear discriminant analysis effect size (LEfSe) was applied to identify the groups that exhibited significant differences in relative abundance between different seasons. In addition, bacterial families with significant differences between different forest age areas were statistically identified using STAMP (v2.1.3).

## 3. Results

### 3.1. Environmental Characteristics of Ao River Estuary Mangrove Sediments

The physicochemical properties of Ao River estuary mangrove sediments across four seasons are summarized in Table 1. Sediments were characterized as silty clay [37], with silt composing the highest proportion (54.72% to 79.0%) and clay content ranging from 20.03% to 45.25%. Significant seasonal temperature variations were observed (*p* < 0.05), with the highest average temperatures in summer (30.02 °C) and the lowest in winter (14.39 °C). Salinity and soil water-soluble salt content also exhibited significant seasonal variations, with the lowest salinity in spring (mean 10.79 ppt) and the highest in autumn (mean 29.01 ppt). Conversely, soil water-soluble salt content peaked in spring (46.29 g/kg) and reached its lowest point in summer (10.19 g/kg). The pH exhibited minor fluctuations across seasons and river sections, varying from 6.66 to 8.92, which indicates weakly alkaline conditions generally. The total carbon (TC) and total nitrogen (TN) contents showed minimal spatial and temporal variation (9.05 to 15.78 g/kg and 0.55 to 1.41 g/kg, respectively). The carbon-to-nitrogen (C/N) ratio averaged 13.71 in winter, which was significantly higher than in other seasons.

### 3.2. Bacterial Diversity of Ao River Estuary Mangrove Sediments

**Figure 2:**
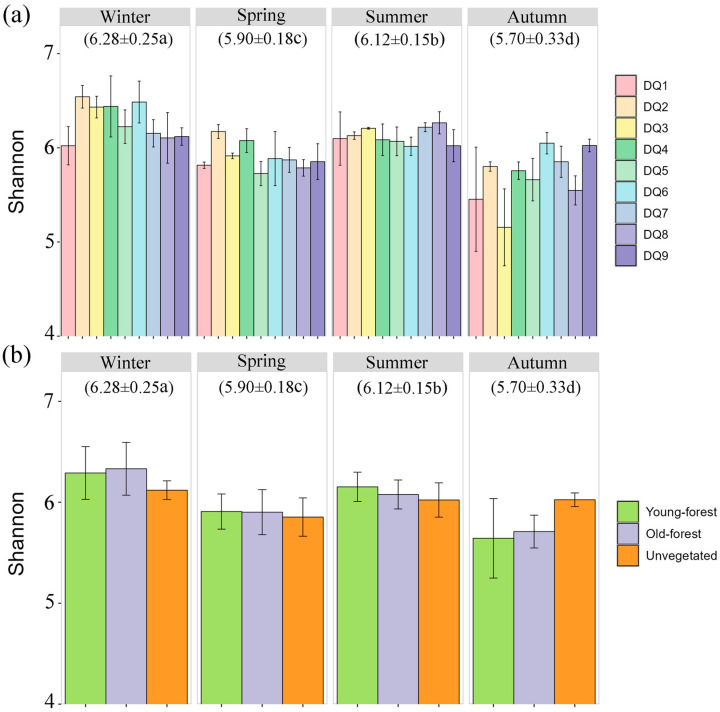
Shannon indices of bacterial communities in each section (**a**) and different forest area (**b**) in different seasons. The numbers in parentheses indicate the average value. a–d indicate statistically significant differences (*p* < 0.05).

A total of 6,940,249 high-quality sequences were obtained and clustered into 19,290 OTUs at a 97% similarity threshold. Sequencing depth, as indicated by the dilution curves (Figures S1 and S2), adequately captured the bacterial communities of sediment samples. The Shannon index, which is indicative of microbial community stability and diversity, showed significant seasonal variation, with the highest average in winter (6.28 ± 0.25) and the lowest in autumn (5.70 ± 0.33) (Figure 2a). Spatial variation was also observed, with upstream sections (DQ1–DQ4) exhibiting significantly higher Shannon indices than mid–lower reaches (DQ5–DQ9) during winter and spring, with the opposite pattern observed during summer and autumn. However, the Shannon indices showed no significant differences between different forest areas (young forest, old forest, and unvegetated control). (Figure 2b). Young forest generally exhibited the highest Shannon index values across all seasons, with notable peaks in winter and summer. In contrast, the unvegetated sediments exhibited lower diversity compared to forested sediments, with a particularly sharp decline in autumn. The old forest sediments maintained intermediate levels of diversity, with less pronounced seasonal fluctuations.

**Figure 3:**
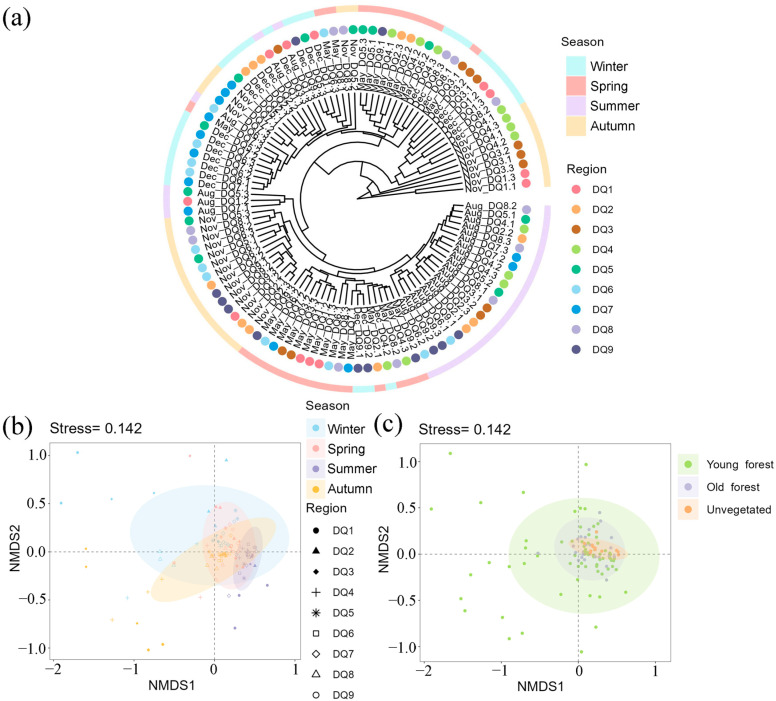
The clustering analysis of bacterial communities in each section in different seasons (**a**); NMDS analysis of bacterial communities in each section in different seasons (**b**) and in different forest areas (**c**). The color circle areas in NMDS analysis represent the 95% confidence interval.

Cluster analysis revealed that most of the summer samples formed a distinct branch, with some spring and autumn samples also clustering together, indicating a high similarity among bacterial communities in these seasons (Figure 3a). Similar to the results of cluster analysis, NMDS analysis based on bacterial OTUs showed that the samples were most tightly clustered in summer, followed by spring and autumn, while winter samples were more dispersed (Figure 3b). An obvious overlap between sample spots from different seasons suggested a degree of stability in the sediment bacterial community structures of the Ao River estuary mangroves, although seasonal heterogeneity was evident, likely influenced by environmental variations within the estuary.

The NMDS illustrated clear clustering of microbial communities based on sediment type (young forest, old forest, and unvegetated) (Figure 3c). Young forest sediments exhibited the widest dispersion, indicating greater variability in community composition. Old forest sediments formed a tighter cluster, reflecting a more uniform community composition. The unvegetated sediments also displayed a distinct cluster, suggesting that the presence of mangrove vegetation significantly impacts microbial community structure. Overlapping confidence ellipses suggested some degree of compositional similarity, yet the distinct separation of group centroids highlighted the unique microbial communities associated with each sediment type [38].

### 3.3. Bacterial Community Characteristics by Season and Plants’ Age

**Figure 4:**
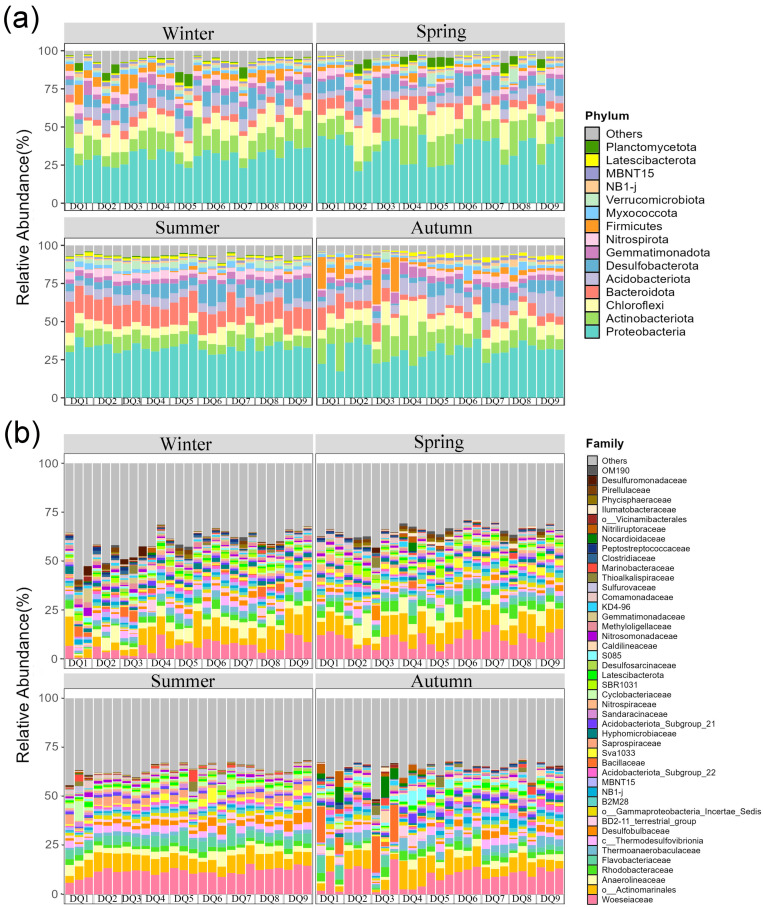
Relative abundances of sediment bacterial community composition from Ao River estuary at the phylum-level (**a**) and family-level (**b**).

Dominant bacterial phyla in the Ao River estuary mangrove sediments remained largely consistent across seasons with minor abundance variations (Figure 4a). Phyla with relative abundances exceeding 5% in winter were Proteobacteria (30.89%), Actinobacteria (12.68%), Chloroflexi (10.67%), Acidobacteria (6.91%), and Desulfobacterota (6.32%). In spring, the dominant phyla were Proteobacteria (35.50%), Actinobacteria (14.26%), Chloroflexi (10.48%), Desulfobacterota (7.03%), Bacteroidetes (5.51%), and Acidobacteria (5.49%); in summer, they were Proteobacteria (33.33%), Bacteroidetes (14.74%), Actinobacteria (8.60%), Desulfobacterota (7.86%), Acidobacteria (5.85%), and Chloroflexi (5.85%); in autumn, they comprised Proteobacteria (30.43%), Actinobacteria (13.43%), Chloroflexi (9.98%), Acidobacteria (8.82%), Desulfobacterota (5.76%), Bacteroidetes (5.60%), and Firmicutes (5.34%). Seasonal heterogeneity was observed in phylum-level community composition, with Proteobacteria, Actinobacteria, Chloroflexi, Bacteroidetes, Acidobacteria, and Desulfobacterota consistently being dominant. The relative abundance of Bacteroidetes exceeded 5% in spring, summer, and autumn, but was lower in winter. Conversely, the relative abundance of Chloroflexi was nearly double in spring and winter compared to summer. Firmicutes were dominant in autumn but comprised less than 5% in other seasons.

The top 45 most abundant bacterial families are shown in Figure 4b. Woeseiaceae and Actinomarinales were consistently dominant across all seasons, with average abundances of 9.27% and 8.07%, respectively. Significant seasonal variations were observed in other bacterial families. For instance, the relative abundance of Bacillaceae was significantly higher in autumn, while Anaerolineaceae was more abundant in spring and winter. In addition, the relative abundances of Flavobacteriaceae and Saprospiraceae (Bacteroidetes) peaked in summer.

**Figure 5:**
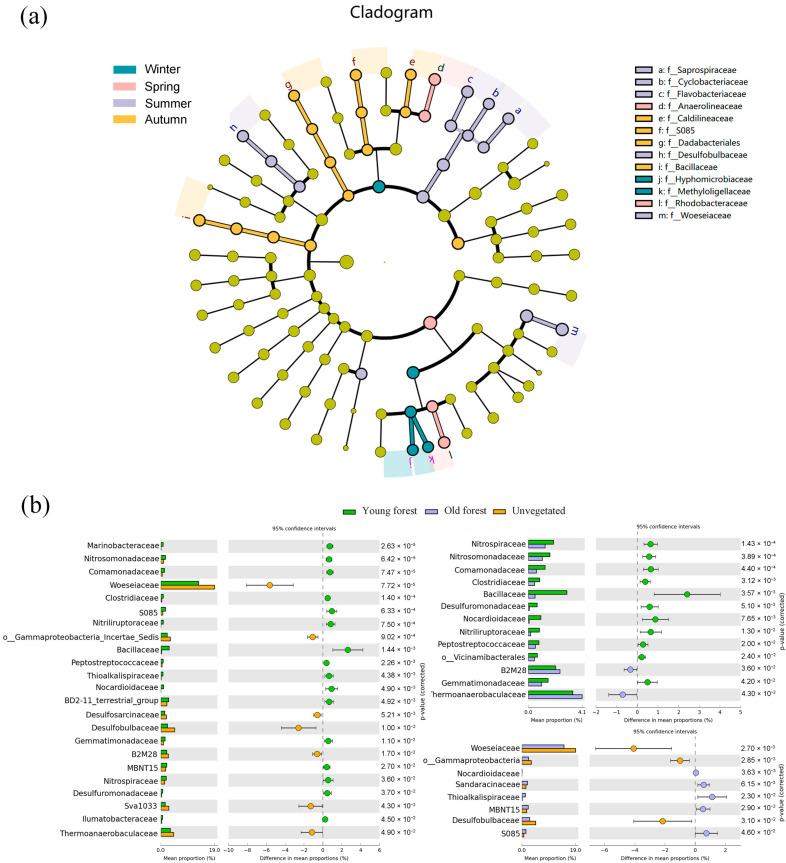
Lefse analysis of bacterial communities in sediments by season (**a**); significantly different bacterial families (top 45) in sediments across different forest areas based on STAMP analysis (**b**). Color circle dots in STAMP analysis indicate the 95% confidence intervals.

There were 13 bacterial families exhibiting significant seasonal differences in LEfSe analysis (Figure 5a). Summer was significantly enriched in Woeseiaceae, Desulfobulbaceae, Flavobacteriaceae, Cyclobacteriaceae, and Saprospiraceae. Autumn was characterized by Bacillaceae, S085, and Caldilineaceae; winter was characterized by Methyloligellaceae and Hyphomicrobiaceae; and spring was characterized by Rhodobacteraceae and Anaerolineaceae. Additionally, STAMP analysis of the top 45 bacterial families revealed significant differences in 24 families between different forest areas, with statistical comparisons performed using ANOVA (Figure 5b). Woeseiaceae, Desulfobulbaceae, Thermoanaerobacteraceae, and Sva1033 exhibited significantly higher relative abundances in the unvegetated area, while Bacillaceae and S085 were more abundant in the mangrove areas. A comparison of young and old forest sediments revealed that Bacillaceae, Nocardioidaceae, and Nitriliruptoraceae exhibited significantly higher relative abundances in young forest sediments, whereas Thermoanaerobacteraceae and B2M28 had significantly higher relative abundances in old forest sediments.

**Figure 6:**
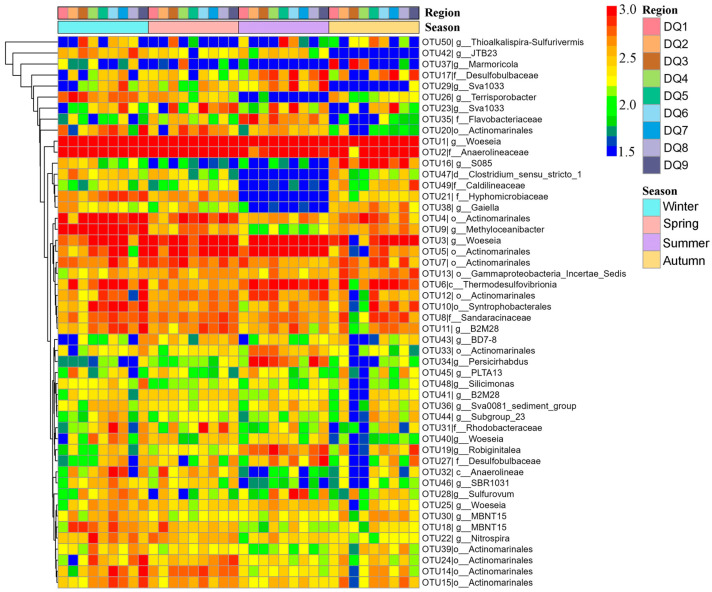
Heatmap of the top 50 OTUs of bacterial community component in Ao River estuary mangrove sediments.

Heatmap clustering of the top 50 OTUs revealed the obvious seasonal differences (Figure 6). In spring, certain OTUs exhibited higher relative abundance. For example, OTU27 (Desulfobulbaceae) and OTU35 (Flavobacteriaceae) showed significantly higher abundance in multiple regions (DQ1, DQ2, and DQ9). However, OTU26 (Terrisporobacter) and OTU38 (Gaiella) had relatively lower abundance in spring. Summer was one of the seasons with the most pronounced microbial community shifts. The heatmap showed that the Desulfobulbaceae (OTU17, OTU27, and OTU29) and the Bacteroidetes (OTU19 and OTU35) exhibited exceptionally high abundance in summer, particularly in the DQ1, DQ2, and DQ9 regions. In contrast, OTUs like Anaerolineae (OTU32) showed a marked decrease in abundance during summer. Additionally, OTU26 (Terrisporobacter) from Firmicutes and OTU38 (Gaiella) from Actinobacteria also exhibited lower abundance in summer.

The OTU distribution in autumn presented a transitional state, with some OTUs having abundance levels between those observed in spring and summer. For instance, OTU17 (Desulfobulbaceae) and OTU35 (Flavobacteriaceae) maintained relatively high abundance in autumn, though slightly lower than in summer. Meanwhile, OTU32 (Anaerolineae) showed an increase in abundance during autumn, but still remained lower than in spring. Furthermore, OTU26 (Terrisporobacter) and OTU38 (Gaiella) exhibited some recovery in autumn, although their abundance had not reached the spring levels. The OTU distribution in winter differed from that in other seasons. Certain OTUs, such as OTU17 (Desulfobulbaceae), showed a significant decrease in abundance during winter, with abundance being approximately 50% of that observed in other seasons. In contrast, OTU32 (Anaerolineae) and OTU46 (SBR1031) had relatively higher abundance in winter, with both being five times more abundant than in summer, which may be related to specific environmental conditions during this season. These results indicated that seasonal variations played an important regulatory role in the composition and function of microbial communities.

### 3.4. Impacts of Environmental Parameters on Bacterial Community and Functional Prediction Analysis

**Figure 7:**
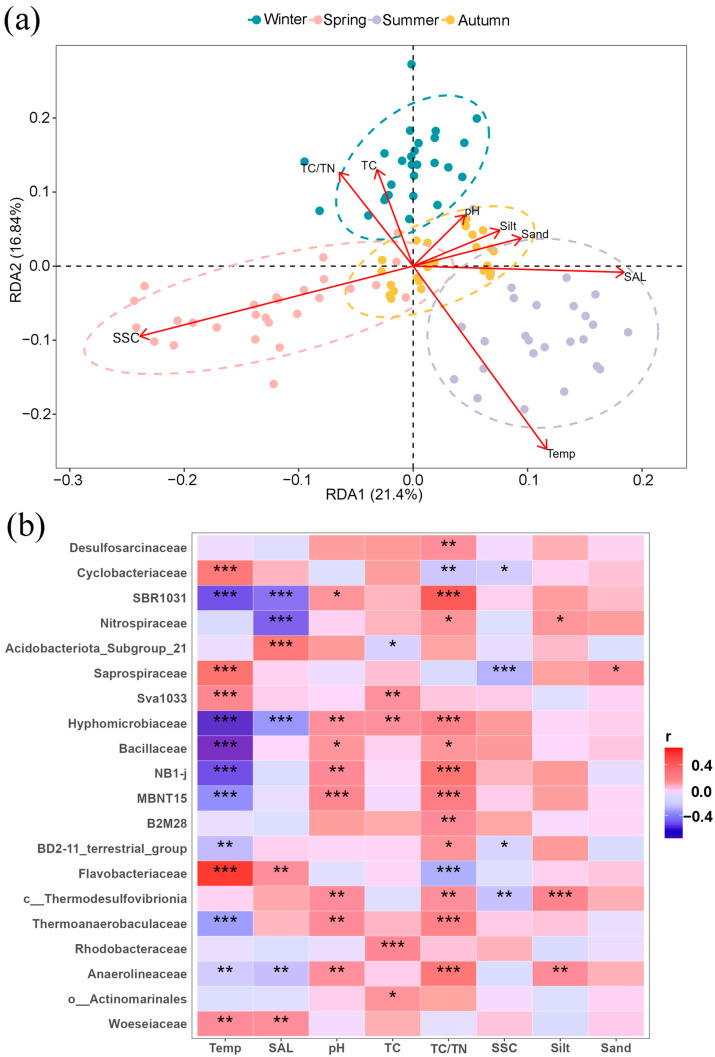
RDA analysis indicated the impacts of environmental parameters on bacterial community structures (**a**); correlation analysis between environmental factors and the relative abundances of bacterial families (**b**). Dotted circles in RDA analysis indicate the 95% confidence intervals. Note: Temp (temperature), SAL (salinity), TC (total carbon), TC/TN (carbon–nitrogen ratio), SSC (soluble salt content), Silt (silt content), and Sand (sand content); * means *p* < 0.05, ** means *p* < 0.01, *** means *p* < 0.001.

RDA analysis (Table S2) revealed that environmental factors, such as temperature and salinity, significantly affected the microbial community of the Ao River estuary after removing TN and clay content due to collinearity. Temperature exhibited a positive correlation with bacterial community structures in summer and a negative correlation in winter (Figure 7a). Salinity exhibited a similar trend, with positive correlation in summer and negative correlation in spring. Furthermore, temperature, pH, TC/TN ratio, and salinity exhibited numerous significant correlations with many bacterial families (Figure 7b), similar to the result of RDA analysis. Flavobacteriaceae, Saprospiraceae, and Cyclobacteriaceae (Bacteroides) were positively correlated with temperature, while being negatively correlated with TC and TC/TN ratio. Anaerolineaceae and SBR1031 showed negative correlations with temperature and salinity, but positive correlations with pH and TC/TN ratio. In contrast, both Bacillaceae (Firmicutes) and Thermoanaerobaculaceae (Actinomycetes) were positively correlated with pH and TC/TN but negatively correlated with temperature. Actinomarinales correlated positively only with TC. Finally, the Woeseiaceae (Proteobacteria) showed significant positive correlations only with temperature and salinity.

PICRUSt2 analysis predicted the functional composition of sediment bacterial communities, identifying six primary functional categories at KEGG level 1 and 46 functional categories at level 2 (Figure S3). Carbohydrate metabolism and amino acid metabolism were particularly abundant, with the average relative abundances of 8.70% and 7.69%, respectively, exceeding 5% in all samples. Energy metabolism (4.75%) and metabolism of cofactors and vitamins (4.47%) also showed relative abundances approaching 5%. These active metabolic functions suggest that the bacterial communities in Ao River estuary mangrove sediments are vigorously engaged in biological activities.

## 4. Discussion

Microbial diversity within mangrove wetland ecosystems plays a critical role in maintaining the ecological balance [6]. In this study, the Shannon indices ranged from 5.16 to 6.54 (Table S1), which were comparable to those of natural mangroves. Tong et al. (2019) found through Illumina MiSeq sequencing that the Shannon index of mangrove sediments in southern China ranged from 4.92 to 5.63 [39]. Loganathachetti et al. (2016) found that the Shannon index of bacterial communities in mangrove plant rhizospheres was around 5.5, indicating high bacterial diversity in mangrove sediments [40]. The Ao River estuary mangroves are located in the estuary area, which is a unique transition zone between land and sea, where freshwater from the river mixes with seawater. This mixing creates a complex and dynamic environment with a wide range of salinity gradients [41]. Different bacterial species have varying tolerances to salinity, and this diverse salinity environment provides suitable habitats for a large number of bacteria with different salt-adaptation abilities, thus promoting the co-existence of various bacterial taxa [42,43]. Moreover, the hydrodynamics of the estuary are complex. Tidal fluctuations, river flows, and water currents constantly reshape the sediment environment [44]. The continuous movement of water can create different micro-habitats, such as areas with different levels of oxygen availability, which further enhances the diversity of bacterial communities [45].

The soil microorganisms in coastal wetland ecosystems have unique functions and genetic profiles, playing an important role in pollutant purification and maintaining ecosystem stability [46]. According to functional predictions using PIRCUSt, the microbial community in the sediment of the Ao River estuary mangroves is rich in functions, with the most active pathways being carbohydrate metabolism, amino acid metabolism, energy metabolism, cofactor and vitamin metabolism, and translation. Carbohydrates are the main component of living cell structures and also serve as substrates for cellular respiration. Carbohydrate metabolism regulates the formation, decomposition, and interconversion of carbohydrates within organisms [47]. Meanwhile, cofactor and vitamin metabolism, as well as amino acid metabolism, are closely related to the degradation of various amino acids [48]. The high abundance of these metabolic functional genes indicates that the bacterial community in the sediment of the Ao River estuary mangroves is highly active.

Aligning with the findings of other studies, Proteobacteria emerged as the phylum with the highest relative abundance in this study [49,50,51]. As Gram-negative bacteria, Proteobacteria widely participate in nitrogen fixation and nutrient cycling in natural environments, suggesting that their abundances in mangroves may be related to the high rates of material and energy metabolism present in these ecosystems [5]. The dominance of Proteobacteria across all seasons can likely be attributed to their metabolic diversity and adaptability. These bacteria can efficiently utilize a wide range of organic compounds and play key roles in various biogeochemical processes, such as nitrogen cycling and organic matter degradation. This versatility is particularly important in the dynamic mangrove environment, where temperature, humidity, and nutrient availability fluctuate with seasonal changes [52]. Other high-abundance phyla observed in this study, including Actinobacteria, Chloroflexi, Bacteroidetes, Acidobacteria, and Desulfobacterota, have also been frequently observed in other mangrove sediments [49,50]. However, the dominant phyla exhibited differences in relative abundance among these studies. For example, Actinobacteria was the second most abundant phylum in this research, accounting for 12.28% of total sequences, whereas it comprised only 7% in Indian mangrove samples [51]. In contrast, Planctomycetes represented 7.2% of samples from Hong Kong’s mangrove sediments, but only accounted for 1.15% in Ao River estuary mangroves [49]. Overall, both this study and previous studies showed that Proteobacteria was the most abundant in mangrove sediments, and that the other dominant phyla were largely consistent.

In this study, bacterial diversity in the mangrove sediments of the Ao River estuary exhibited obvious seasonal variations. The Shannon index was the highest in winter among the seasons, significantly exceeding the values observed in spring and summer. Previous research has indicated that the duration of daylight is significantly associated with marine bacterial richness, as shorter photosynthetic cycles contribute to increased bacterial diversity [53]. Intertidal sediments are frequently exposed to air, and light influences directly affect microbial communities. In the northern hemisphere, shorter daylight periods during winter lead to reduced photosynthetic cycles, which primarily contribute to the increased bacterial diversity observed in winter compared to spring and summer. Furthermore, Ladau’s study showed that microbial diversity in temperate oceans peaks during the winter months [54]. The mangrove forest in the Ao River estuary is situated in a subtropical region at the interface of land and sea, where it is repeatedly influenced by the tidal actions, making it particularly susceptible to the marine environment. These factors likely explain why the Shannon index of bacterial communities in mangrove sediments of the Ao River estuary was higher in winter.

Additionally, bacterial community compositions also exhibited seasonal disparities. For instance, Woeseiaceae, which demonstrated the highest relative abundance of bacterial families, was significantly more prevalent in summer, correlating to peak salinity and temperature levels, consistent with previous studies [55]. Woeseiaceae, classified within the γ-Proteobacteria, has been previously reported to correlate strongly with salinity, indicating preference for saline environments [56]. Heterotrophic bacteria, such as Bacteroidetes, were also more abundant in summer than in other seasons. As water temperature rises in summer, the metabolic ability of planktonic bacteria gradually increases. Bacteroidetes contribute to their own growth by degrading organic matter from algae, and the decomposition product by Bacteroidetes can also provide a food source for other heterotrophic bacteria, resulting in a higher abundance during summer compared to other seasons [57]. Temperature plays a crucial role in determining microbial growth rates and biomass, with fluctuations directly influencing microbial metabolic activities, reproduction rates, and community compositions. Studies indicated that elevated temperatures can enhance the growth and metabolic activities of certain bacteria; however, excessive heat may favor autotrophic microorganisms over heterotrophic ones, increasing effective carbon absorption and intensifying nutrient limitations, which can lead to substantial reductions in soil microbial biomass [58].

The relative abundances of Actinomarinales and Thermoanaerobaculaceae were significantly higher in winter than in summer and autumn. Actinomarinales and Thermoanaerobaculaceae belong to Actinobacteria, which play an important role in the carbon cycle by degrading various organic substances such as cellulose, hemicellulose, pectin, and chitin [59]. Previous studies have shown that Actinobacteria are typically found in carbon-rich, near-neutral, or weakly alkaline soils [60]. Therefore, higher total carbon and pH levels may result in increased relative abundances of Actinomarinales and Thermoanaerobaculaceae in winter samples. Bacillus exhibits strong resistance to adverse conditions and has the ability to thrive in nutrient-poor environments [61]. Consequently, during the autumn and winter, when rainfall was limited and nutrients were scarce, Bacillus was better able to adapt to environmental changes. In contrast, the relative abundance of bacteria such as Proteobacteria and Desulfovibrio showed a significant decline, providing Bacillus with a competitive survival advantage [62]. As a result, its relative abundance was higher in autumn and winter and lower in spring and summer.

Salinity constitutes another vital variable influencing the mangrove sediment bacterial community structure. Several bacterial families, such as SBR1031 and Anaerolineaceae, were significantly negatively correlated with salinity, consistent with the negative correlation between salinity and enzyme activity observed in some studies of saline soils [63]. Excessive salinity elevates the osmotic pressure environment, adversely impacting salt-sensitive bacteria by causing desiccation or death. Conversely, excessively low salinity can rupture bacterial cells due to overhydration [64]. As salinity rises, the adsorption of organic matter to sediment substrates diminishes, leading to faster decomposition and loss of organic carbon, ultimately threatening the nutrient conditions vital for bacterial survival and indirectly affecting community structures [65]. Furthermore, increased sediment salinity imposes stress on mangrove plants, consequently affecting the bacterial nutrient sources derived from mangrove root exudates, which may further influence bacterial community configurations [66].

However, when comparing the bacterial communities in the sediments of mangrove areas (both old and young forests) with those in the unvegetated area, Woeseiaceae, Desulfobulbaceae, Thermoanaerobacteraceae, and Sva1033 were found to exhibit significantly higher relative abundances in the unvegetated area, while Bacillaceae and S085 showed higher relative abundances in the mangrove areas (Figure 5b). In other studies, similar differences between mangrove areas and unvegetated areas have also been observed. Sun et al. (2024) found that the Bacillus and Actinobacteria had a much higher relative abundance in the mangrove areas [23]. The reasons for these differences are multi-fold. Mangrove ecosystems provide a complex environment characterized by alternating cycles of high and low tides, as well as gradients of salinity, pH, and nutrient availability, all of which could influence the microbial populations present [67]. In these areas, microbial communities are more likely to include halophilic and salt-tolerant organisms, as well as those adapted to dynamic and nutrient-rich sediment conditions. Bacillaceae may thrive in these conditions due to their spore-forming ability and capacity to withstand fluctuating environmental stressors, such as changes in salinity or moisture [68]. In unvegetated areas, the absence of mangrove might result in higher levels of organic matter decomposition due to reduced plant-driven oxygenation [69]. This could create conditions favorable to bacteria that thrive in anaerobic or low-oxygen environments, such as Desulfobulbaceae [22]. Although no significant differences were observed in bacterial diversity indices across the three mangrove regions, these findings suggest that mangrove cover affects the sediment microbial community structure. However, this effect may be obscured by seasonal variations and other factors [70]. The mangrove ecosystem fosters microbial communities that are adapted to the unique environmental conditions of coastal regions, such as fluctuations in salinity and nutrient gradients, which are vital for nutrient cycling and ecosystem health. The differences in microbial composition between mangrove and unvegetated areas also highlight the role of mangrove plants in modifying sediment conditions and influencing microbial communities, further emphasizing the importance of mangrove ecosystems in coastal ecological processes.

## 5. Conclusions

The bacterial diversity indices in sediment samples from the Ao River estuary mangrove areas across different seasons were all relatively high, with Shannon indices varying between 6.86 and 9.67 and being the highest in winter. In line with previous research, Proteobacteria was the most abundant phylum in the Ao River estuary mangrove sediments, and other high-abundance phyla like Actinobacteria, Chloroflexi, Bacteroidetes, Acidobacteria, and Desulfobacterota were also commonly found in the mangrove sediments.

There were significant seasonal changes in the bacterial diversity and community composition of mangrove sediments in the Ao River estuary. In terms of bacterial diversity, the Shannon index was the highest in winter, which was significantly higher than that in spring and summer. This may be due to the short days in the northern hemisphere in winter, the reduction of the photosynthetic cycle, and the impact of marine environment. In terms of community composition, different bacterial groups have different relative abundances in different seasons. The heterotrophic bacteria such as woeseiaceae and bacteroidea were more abundant in summer, which is related to the high salinity, high temperature, and the enhanced metabolic ability of planktonic bacteria in summer. The relative abundance of actinomycetes in winter was significantly higher than that in summer and autumn, which may be related to the higher total carbon and pH levels in winter samples. The relative abundance of Bacillus was high in autumn and winter because it can adapt to the environment of less rainfall and nutrient deficiency, and at those times, the relative abundance of bacteria such as Proteus and Desulfovibrio decreased, which provided a competitive advantage. Comparing the bacterial communities in sediments of mangrove areas (old forest and young forest) and the unvegetated area, the relative abundance of woeseiaceae in the unvegetated area was significantly higher, and the relative abundance of Bacillaceae in mangrove areas was higher. This indicates that the mangrove ecosystem environment is more complex, which is conducive to the survival of halophilic and salt-tolerant organisms and of organisms that adapt to dynamic eutrophic sediment conditions.

These findings are important for improving mangrove restoration strategies. Through understanding seasonal changes in bacterial diversity and community composition well, we can develop more effective restoration methods. For example, identifying bacterial families that thrive in mangrove sediments can help us to design microbial treatments or soil amendments to boost microbial activity in degraded areas. Additionally, restoration efforts that mimic the natural conditions of mangrove ecosystems—such as maintaining salinity and nutrient balance—can help establish healthy, diverse microbial communities, supporting long-term restoration and the overall health of these important coastal ecosystems.

## Figures and Tables

**Table 1:** Physicochemical properties of mangrove sediments in the Ao River estuary.

Parameter	Winter 2021	Spring 2022	Summer 2022	Autumn 2022
Temperature (°C)	14.39 ± 0.62 ^d^	21.97 ± 1.03 ^b^	30.02 ± 0.93 ^a^	21.16 ± 0.71 ^c^
Salinity (ppt)	15.24 ± 5.67 ^c^	10.79 ± 5.47 ^d^	24.64 ± 7.44 ^b^	29.01 ± 2.77 ^a^
pH	7.87 ± 0.31 ^a^	7.54 ± 0.45 ^b^	7.52 ± 0.23 ^b^	7.66 ± 0.29 ^ab^
Total carbon (g/kg)	12.52 ± 0.60 ^a^	12.21 ± 0.88 ^a^	11.97 ± 0.82 ^a^	11.88 ± 0.89 ^a^
Total nitrogen (g/kg)	0.93 ± 0.12 ^a^	0.97 ± 0.13 ^a^	1.01 ± 0.83 ^a^	0.97 ± 0.13 ^a^
Total carbon/total nitrogen (%)	13.71 ± 1.09 ^a^	12.79 ± 1.10 ^b^	12.04 ± 0.69 ^b^	12.29 ± 1.51 ^b^
Soluble salt content (g/kg)	12.59 ± 3.55 ^c^	46.29 ± 26.97 ^a^	10.19 ± 4.91 ^c^	27.39 ± 8.77 ^b^
Clay content (%)	28.88 ± 3.40 ^b^	36.04 ± 5.25 ^a^	29.99 ± 3.85 ^b^	35.08 ± 3.48 ^a^
Silt content (%)	69.12 ± 3.91 ^a^	63.59 ± 4.96 ^b^	67.88 ± 3.86 ^a^	63.67 ± 3.05 ^b^
Sand content (%)	1.99 ± 1.19 ^a^	0.37 ± 0.64 ^b^	2.13 ± 1.29 ^a^	1.25 ± 2.19 ^ab^

a–d indicate statistically significant differences (*p* < 0.05).

## Data Availability

The datasets presented in this study can be found in online repositories. The names of each repository and the accession number can be found in this article and the Supplementary Materials.

## Supplementary Materials

The following supporting information can be downloaded at: https://www.mdpi.com/article/10.3390/microorganisms13040859/s1, Figure S1. Dilution curves of bacterial communities in sediment samples (Richness); Figure S2. Dilution curves of bacterial communities in sediment samples (Shannon); Figure S3. Heatmap showing the distributions of bacterial functions that were assigned by PICRUSt2 across seasons; Table S1. Bacterial community diversity index of mangrove sediments in this study; Table S2. The influence of environmental factors on bacterial communities and the significance assessment in RDA analysis; Table S3. Sedimentary environmental parameters of different seasons at each sampling site in this study.

## References

[1] Yang X., Dai Z., Yuan R., Guo Z., Xi H., He Z., Wei M. (2023). Effects of Salinity on Assembly Characteristics and Function of Microbial Communities in the Phyllosphere and Rhizosphere of Salt-Tolerant Avicennia marina Mangrove Species. Microbiol. Spectr..

[2] Sandilyan S., Kathiresan K. (2012). Mangrove conservation: A global perspective. Biodivers. Conserv..

[3] Tang J., Ye S., Chen X., Yang H., Sun X., Wang F., Wen Q., Chen S. (2018). Coastal blue carbon: Concept, study method, and the application to ecological restoration. Sci. China Earth Sci..

[4] Wang Y.-S., Gu J.-D. (2021). Ecological responses, adaptation and mechanisms of mangrove wetland ecosystem to global climate change and anthropogenic activities. Int. Biodeterior. Biodegrad..

[5] Holguin G., Vazquez P., Bashan Y. (2001). The role of sediment microorganisms in the productivity, conservation, and rehabilitation of mangrove ecosystems: An overview. Biol. Fertil. Soils.

[6] Saleem M., Hu J., Jousset A. (2019). More Than the Sum of Its Parts: Microbiome Biodiversity as a Driver of Plant Growth and Soil Health. Annu. Rev. Ecol. Evol. Syst..

[7] Xing Y., Qiu J., Chen J., Cheng D., Yin Q., Chen X., Xu L., Zheng P. (2024). Unveiling hidden interactions: Microorganisms, enzymes, and mangroves at different stages of succession in the Shankou Mangrove Nature Reserve, China. Sci. Total Environ..

[8] Zhuang W., Yu X., Hu R., Luo Z., Liu X., Zheng X., Xiao F., Peng Y., He Q., Tian Y. (2020). Diversity, function and assembly of mangrove root-associated microbial communities at a continuous fine-scale. NPJ Biofilms Microbiomes.

[9] Baker B.J., Lazar C.S., Teske A.P., Dick G.J. (2015). Genomic resolution of linkages in carbon, nitrogen, and sulfur cycling among widespread estuary sediment bacteria. Microbiome.

[10] Rashid M.I., Mujawar L.H., Shahzad T., Almeelbi T., Ismail I.M.I., Oves M. (2016). Bacteria and fungi can contribute to nutrients bioavailability and aggregate formation in degraded soils. Microbiol. Res..

[11] Kwon S.K. (2016). Marine Glycobiology: Principles and Applications.

[12] Tam N.F.Y., Guo C.L., Yau W.Y., Wong Y.S. (2002). Preliminary study on biodegradation of phenanthrene by bacteria isolated from mangrove sediments in Hong Kong. Mar. Pollut. Bull..

[13] Tam N.F.Y. (1998). Effects of wastewater discharge on microbial populations and enzyme activities in mangrove soils. Environ. Pollut..

[14] Al-Sayed H.A., Ghanem E.H., Saleh K.M. (2005). Bacterial community and some physico-chemical characteristics in a subtropical mangrove environment in Bahrain. Mar. Pollut. Bull..

[15] Sun S., Sidhu V., Rong Y., Zheng Y. (2018). Pesticide Pollution in Agricultural Soils and Sustainable Remediation Methods: A Review. Curr. Pollut. Rep..

[16] Silliman B.R., Gedan K.B. (2009). Using Facilitation Theory to Enhance Mangrove Restoration. AMBIO A J. Hum. Environ..

[17] Cong J., Yang Y., Liu X., Lu H., Liu X., Zhou J., Li D., Yin H., Ding J., Zhang Y. (2015). Analyses of soil microbial community compositions and functional genes reveal potential consequences of natural forest succession. Sci. Rep..

[18] Fu X.M., Tang H.Y., Liu Y., Zhang M.Q., Jiang S.S., Yang F., Li X.Y., Wang C.Y. (2021). Resource status and protection strategies of mangroves in China. J. Coast. Conserv..

[19] Wang M., Cao W., Guan Q., Wu G., Wang F. (2018). Assessing changes of mangrove forest in a coastal region of southeast China using multi-temporal satellite images. Estuar. Coast. Shelf Sci..

[20] O’Connell D.P., Fusi M., Djamaluddin R., Rajagukguk B.B., Bachmid F., Kitson J.J.N., Dunnett Z., Trianto A., Tjoa A.B., Diele K. (2021). Assessing mangrove restoration practices using species-interaction networks. Restor. Ecol..

[21] Dar S.A., Kleerebezem R., Stams A.J.M., Kuenen J.G., Muyzer G. (2008). Competition and coexistence of sulfate-reducing bacteria, acetogens and methanogens in a lab-scale anaerobic bioreactor as affected by changing substrate to sulfate ratio. Appl. Microbiol. Biotechnol..

[22] Sun D., Huang Y., Wang Z., Tang X., Ye W., Cao H., Shen H. (2023). Soil microbial community structure, function and network along a mangrove forest restoration chronosequence. Sci. Total Environ..

[23] Aaron M. (2000). Ellison. Mangrove Restoration: Do We Know Enough?. Restor. Ecol..

[24] Pérez-Cobas A.E., Gomez-Valero L., Buchrieser C. (2020). Metagenomic approaches in microbial ecology: An update on whole-genome and marker gene sequencing analyses. Microb. Genom..

[25] Selvaraj S., Kannan M.R., Pattapulavar V., Godwin Christopher J., Sasikumar S., Muthusamy S., Manikkam R., Venugopal G. (2025). Mangrove Microbiome.

[26] Lin G., He Y., Lu J., Chen H., Feng J. (2021). Seasonal variations in soil physicochemical properties and microbial community structure influenced by Spartina alterniflora invasion and *Kandelia obovata* restoration. Sci. Total Environ..

[27] Liu C., Zhuang J., Wang J., Fan G., Feng M., Zhang S. (2022). Soil bacterial communities of three types of plants from ecological restoration areas and plant-growth promotional benefits of *Microbacterium invictum* (strain X-18). Front. Microbiol..

[28] Chen H., Chen T., Cai L., Li Z., Chen S., Zhu Y., Huang X., Li C. (2023). Analysis of Soil Bacterial Commnity Structure Characteristics in Wetland of Riparian Vegetation Buffer Zone in Ao River Basin, Zhejiang Province. Acta Agric. Jiangxi.

[29] Chen Q. (2019). Development history and discussion of mangrove forest in Zhejiang Province. Zhejiang Agric. Sci..

[30] Yu Y., Shui B.-N., Lú C.-C., Li B., Li X.-L., Wei Z., Hu C.-Y. (2024). Analysis of the Characteristics and Sources of Organic Carbon Burial in Mangrove Wetland Sediments of Maoyan Island. China Environ. Sci..

[31] Faé S.G., Montes F., Bazilevskaya E., Añó R.M., Kemanian A.R. (2019). Making Soil Particle Size Analysis by Laser Diffraction Compatible with Standard Soil Texture Determination Methods. Soil Sci. Soc. Am. J..

[32] Shang S., Hu S., Liu X., Zang Y., Chen J., Gao N., Li L., Wang J., Liu L., Xu J. (2022). Effects of Spartina alterniflora invasion on the community structure and diversity of wetland soil bacteria in the Yellow River Delta. Ecol. Evol..

[33] Jiang C., Diao X., Wang H., Ma S. (2021). Diverse and abundant antibiotic resistance genes in mangrove area and their relationship with bacterial communities—A study in Hainan Island, China. Environ. Pollut..

[34] Michael H., Beiko R.G. (2018). 16S rRNA Gene Analysis with QIIME2. Methods Mol. Biol..

[35] Balvočiūtė M., Huson D.H. (2017). SILVA, RDP, Greengenes, NCBI and OTT—How do these taxonomies compare?. BMC Genom..

[36] Peng X., Guo F., Ju F., Zhang T. (2014). Shifts in the microbial community, nitrifiers and denitrifiers in the biofilm in a full-scale rotating biological contactor. Environ. Sci. Technol..

[37] Wang L., Jian X., Mei H., Shen X., Fu H. (2024). Clay composition heterogeneity in sediments from mountainous catchments with contrasting bedrock lithology in SE China coast. Catena.

[38] Ramette A. (2007). Multivariate analyses in microbial ecology. FEMS Microbiol. Ecol..

[39] Tong T., Li R., Wu S., Xie S. (2019). The distribution of sediment bacterial community in mangroves across China was governed by geographic location and eutrophication. Mar. Pollut. Bull..

[40] Sanka Loganathachetti D., Sadaiappan B., Poosakkannu A., Muthuraman S. (2016). Pyrosequencing-Based Seasonal Observation of Prokaryotic Diversity in Pneumatophore-Associated Soil of *Avicennia marina*. Curr. Microbiol..

[41] Chang Y., Li X., Wang P.Y., Klingbeil K., Li W., Zhang F., Burchard H. (2024). Salinity mixing in a tidal multi-branched estuary with huge and variable run off. J. Hydrol..

[42] Rath K.M., Fierer N., Murphy D.V., Rousk J. (2019). Linking bacterial community composition to soil salinity along environmental gradients. ISME J..

[43] Chen Z., Zhou T., Huang G., Xiong Y. (2024). Soil microbial community and associated functions response to salt stresses: Resistance and resilience. Sci. Total Environ..

[44] Zhang F., Sun J., Lin B., Huang G. (2018). Seasonal hydrodynamic interactions between tidal waves and river flows in the Yangtze Estuary. J. Mar. Syst..

[45] Guo Z., Li Y., Shao M., Sun T., Lin M., Zhang T., Hu K., Jiang H., Guan X. (2023). Succession and environmental response of sediment bacterial communities in the Liao River Estuary at the centenary scale. Mar. Environ. Res..

[46] Xie X.F., Xiang Q., Wu T., Jiang G.J., Sun X.M., Zhu M., Pu L.J. (2021). Progress and prospect of soil microorganisms and their influencing factors in coastal wetland ecosystem. Acta Ecol. Sin..

[47] Sun X., Lin Y.-L., Li B.-L., Huang L.-F. (2020). Analysis and function prediction of soil microbial communities of *Cynomorium songaricum* in two daodi-origins. Acta Pharm. Sin..

[48] Miao L.Z., Wang P.F., Hou J., Yao Y., Liu Z., Liu S., Li T. (2019). Distinct community structure and microbial functions of biofilms colonizing microplastics. Sci. Total Environ..

[49] Jiang X.T., Peng X., Deng G.H., Sheng H.F., Wang Y., Zhou H.W., Tam N.F.Y. (2013). Illumina Sequencing of 16S rRNA Tag Revealed Spatial Variations of Bacterial Communities in a Mangrove Wetland. Microb. Ecol..

[50] Basak P., Pramanik A., Sengupta S., Nag S., Bhattacharyya A., Roy D., Pattanayak R., Ghosh A., Chattopadhyay D., Bhattacharyya M. (2016). Bacterial diversity assessment of pristine mangrove microbial community from Dhulibhashani, Sundarbans using 16S rRNA gene tag sequencing. Genom. Data.

[51] Satyanarayana T., Das S.K., Johri B.N. (2019). Microbial Diversity in Ecosystem Sustainability and Biotechnological Applications.

[52] Zhou Z., Tran P.Q., Kieft K., Anantharaman K. (2020). Genome diversification in globally distributed novel marine Proteobacteria is linked to environmental adaptation. ISME J..

[53] Ghiglione J.F., Murray A.E. (2012). Pronounced summer to winter differences and higher wintertime richness in coastal Antarctic marine bacterioplankton. Environ. Microbiol..

[54] Ladau J., Sharpton T., Finucane M., Jospin G., Kembel S.W., O’Dwyer J., Koeppel A.F., Green J.L., Pollard K.S. (2013). Global marine bacterial diversity peaks at high latitudes in winter. ISME J..

[55] Ma J., Zhou T., Xu C., Shen D., Xu S., Lin C. (2020). Spatial and Temporal Variation in Microbial Diversity and Community Structure in a Contaminated Mangrove Wetland. Appl. Sci..

[56] Klier J., Dellwig O., Leipe T., Jürgens K., Herlemann D.P.R. (2018). Benthic Bacterial Community Composition in the Oligohaline-Marine Transition of Surface Sediments in the Baltic Sea Based on rRNA Analysis. Front. Microbiol..

[57] Xue J., Xiao N., Wang Q., Wu H. (2016). Seasonal variation of bacterial community diversity in Yangshan Port area. Acta Ecol. Sin..

[58] Liu Y., Gao K., Guo Z., Liu X., Bian R., Sun B., Li J., Chen J. (2022). An antagonistic effect of elevated CO_2_ and warming on soil N_2_O emissions related to nitrifier and denitrifier communities in a Chinese wheat field. Plant Soil.

[59] Bhatti A.A., Haq S., Bhat A.R. (2017). Actinomycetes benefaction role in soil and plant health. Microb. Pathog..

[60] Dang P., Li C., Lu C., Zhang M., Huang T., Wan C., Wang H., Chen Y., Qin X., Liao Y. (2022). Effect of fertilizer management on the soil bacterial community in agroecosystems across the globe. Agric. Ecosyst. Environ..

[61] Collins P.D., Jacobsen J.B. (2003). Optimizing a Bacillus subtilis isolate for biological control of sugar beet cercospora leaf spot. Biol. Control..

[62] An X., Wang Z., Teng X., Zhou R., Wang X., Xu M., Lian B. (2022). Rhizosphere bacterial diversity and environmental function prediction of wild salt-tolerant plants in coastal silt soil. Ecol. Indic..

[63] Rietz D., Haynes R. (2003). Effects of irrigation-induced salinity and sodicity on soil microbial activity. Soil Biol. Biochem..

[64] Estrelles E., Biondi E., Galiè M., Mainardi F., Hurtado A., Soriano P. (2015). Aridity level, rainfall pattern and soil features as key factors in germination strategies in salt-affected plant communities. J. Arid. Environ..

[65] Yan N., Marschner P., Cao W., Zuo C., Qin W. (2015). Influence of salinity and water content on soil microorganisms. Int. Soil Water Conserv. Res..

[66] Boyrahmadi M., Raiesi F. (2018). Plant roots and species moderate the salinity effect on microbial respiration, biomass, and enzyme activities in a sandy clay soil. Biol. Fertil. Soils.

[67] Chen Q., Zhao Q., Li J., Jian S., Ren H. (2016). Mangrove succession enriches the sediment microbial community in South China. Sci. Rep..

[68] Mandic-Mulec I., Stefanic P., van Elsas J.D. (2015). Ecology of Bacillaceae. Microbiol. Spectr..

[69] Zhang Z., Hu L., Liu Y., Guo Y., Tang S., Ren J. (2025). Land use shapes the microbial community structure by altering soil aggregates and dissolved organic matter components. J. Integr. Agric..

[70] Colares B.G., Melo M.M.V. (2013). Relating microbial community structure and environmental variables in mangrove sediments inside *Rhizophora mangle* L. habitats. Appl. Soil Ecol..

