# Supplementary material for: Spatiotemporal Characteristics of Bacterial Communities in Estuarine Mangrove Sediments in Zhejiang Province, China
Source: Microorganisms. 2025 Apr 9;13(4):859. doi: 10.3390/microorganisms13040859 (PMC12029902; doi:10.3390/microorganisms13040859)
Supplement: Supplementary file 1 [file microorganisms-13-00859-s001.zip › Supplementary_Materials.pdf]

Supplementary Materials

1 Supplementary Data

All the original sequences used in the article have been uploaded to the NCBI official website with the serial number PRJNA1137318.

2 Supplementary Figures

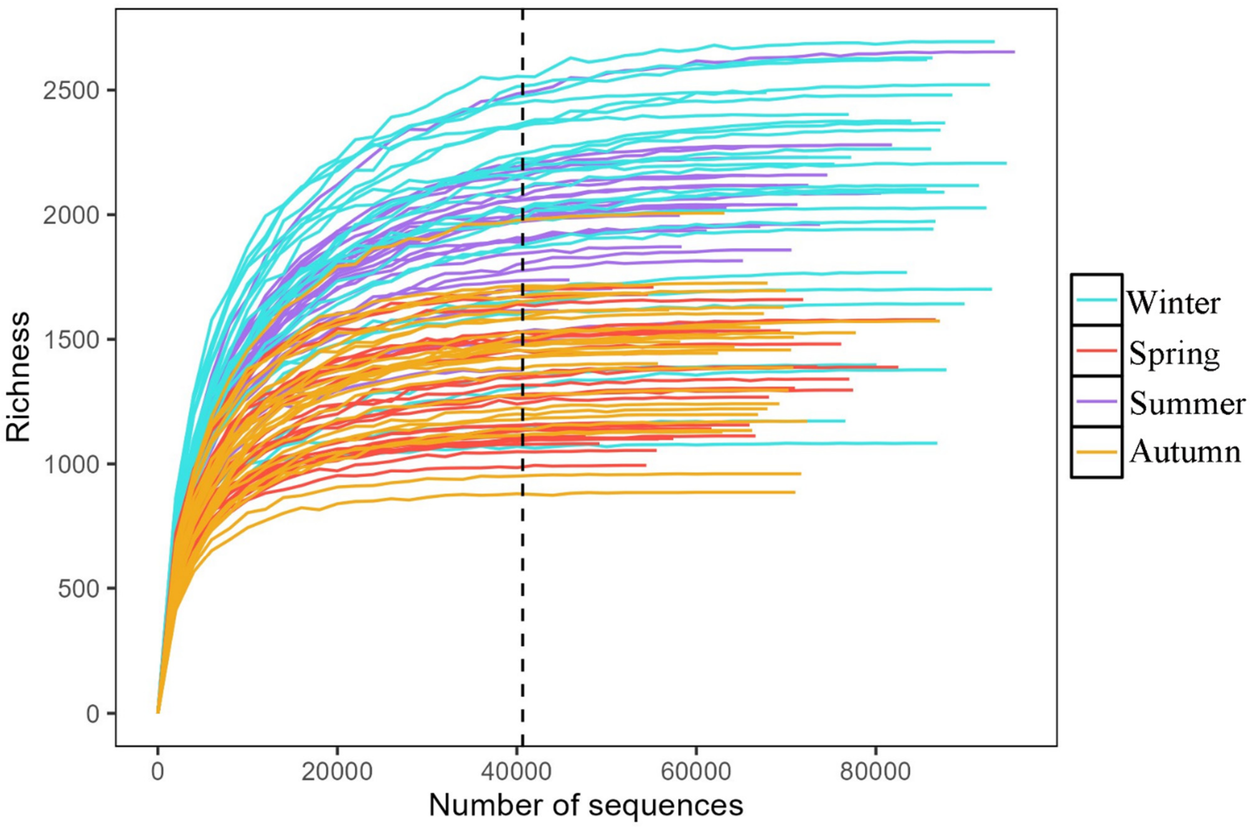

Figure S1. Dilution curves of bacterial communities in sediment samples (Richness)

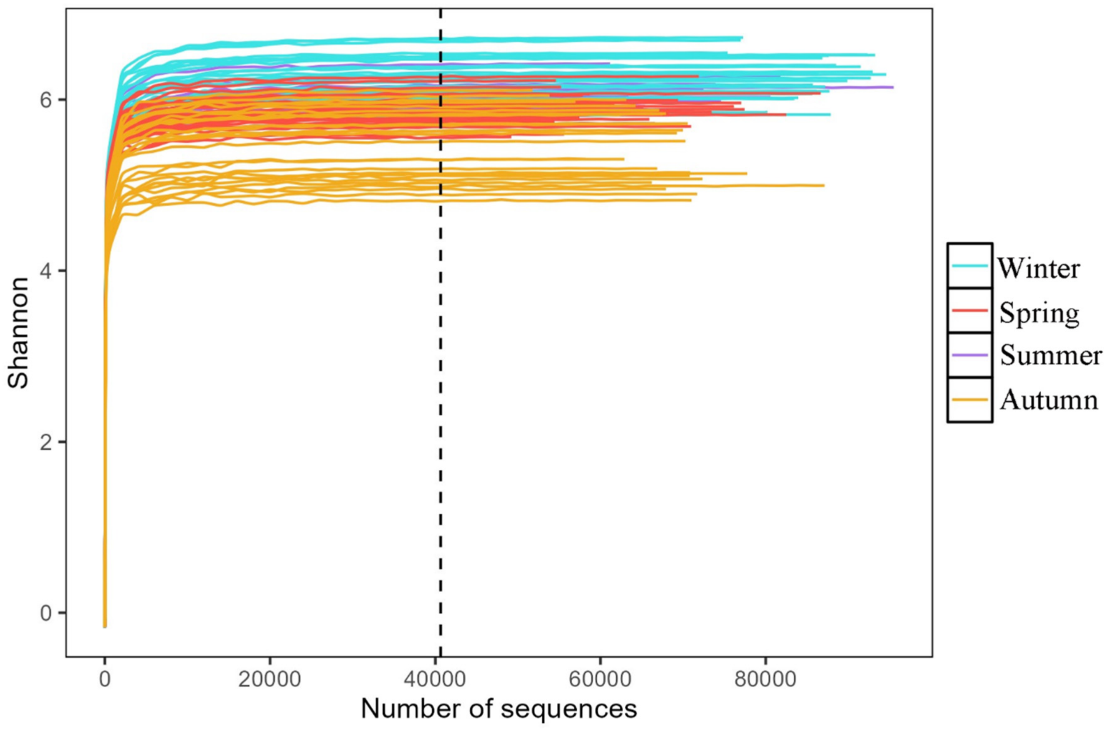

Figure S2. Dilution curves of bacterial communities in sediment samples (Shannon)

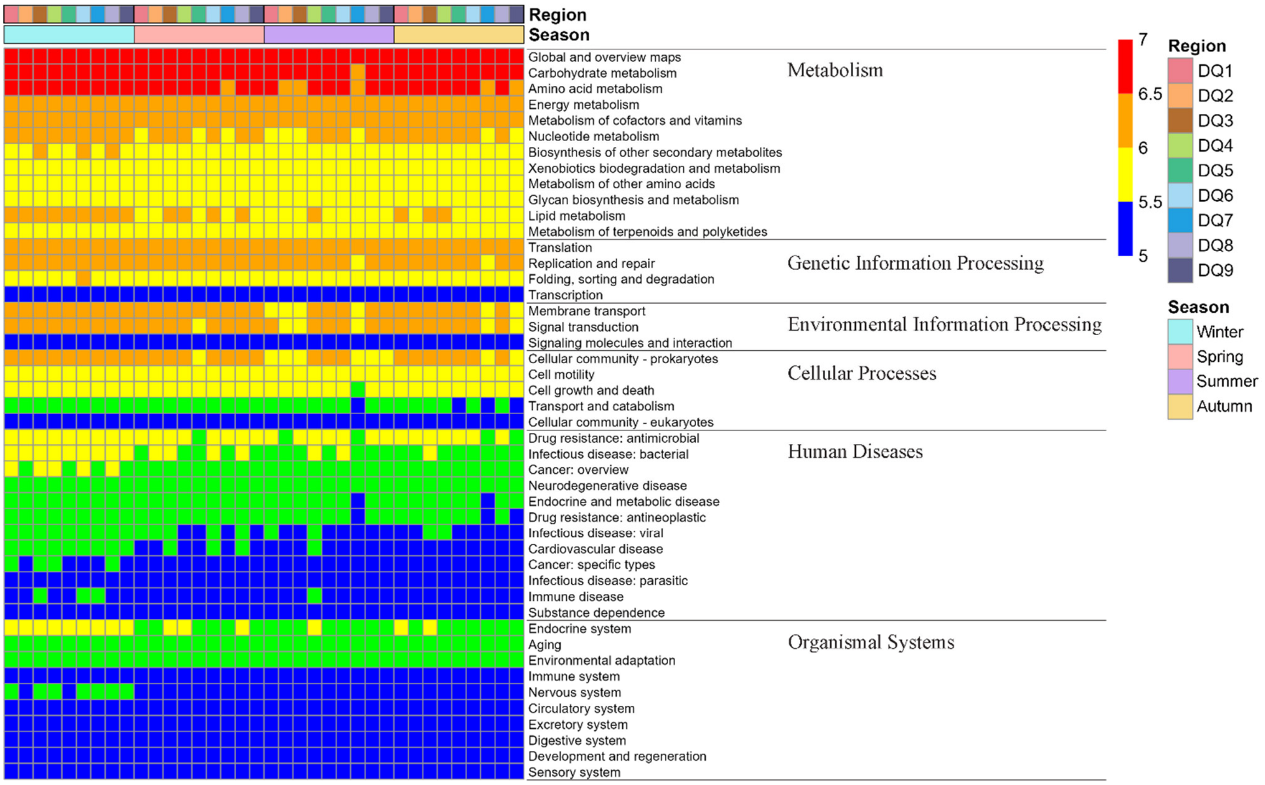

Figure S3. Heatmap showing the distributions of bacterial functions that were assigned by PICRUSt2 across season

3 Supplementary Tables

Table S1. Bacterial community diversity index of mangrove sediments in this study

Season	Sample	Richness	Shannon	Simpson	Pielou	goods_coverage
Winter	DQ1-M1	2009	6.055	0.993	0.552	1
	DQ1-M2	1141	6.206	0.996	0.611	1
	DQ1-M3	1359	5.804	0.993	0.558	1
	DQ2-M1	1960	6.490	0.995	0.593	1
	DQ2-M2	1060	6.456	0.997	0.642	1
	DQ2-M3	2399	6.679	0.996	0.595	1
	DQ3-M1	2030	6.484	0.996	0.590	1
	DQ3-M2	1659	6.300	0.996	0.589	1
	DQ3-M3	2484	6.512	0.994	0.577	1
	DQ4-M1	1341	5.829	0.992	0.561	1
	DQ4-M2	2232	6.120	0.989	0.550	1
	DQ4-M3	2059	6.366	0.995	0.578	1
	DQ5-M1	2148	6.697	0.996	0.605	1
	DQ5-M2	2125	6.546	0.994	0.592	1

	DQ5-M3	2185	6.075	0.992	0.548	1
	DQ6-M1	2350	6.400	0.993	0.572	1
	DQ6-M2	2391	6.229	0.992	0.555	1
	DQ6-M3	1884	6.043	0.992	0.555	1
	DQ7-M1	2539	6.491	0.994	0.574	1
	DQ7-M2	2300	6.705	0.995	0.600	1
	DQ7-M3	2120	6.261	0.994	0.567	1
	DQ8-M1	1604	6.186	0.994	0.581	1
	DQ8-M2	1700	5.995	0.992	0.559	1
	DQ8-M3	1896	6.280	0.995	0.577	1
	DQ9-M1	2186	6.048	0.989	0.545	1
	DQ9-M2	2397	6.224	0.991	0.554	1
	DQ9-M3	2250	6.087	0.992	0.547	1
Spring	DQ1-M1	1070	5.816	0.990	0.578	1
	DQ1-M2	1065	5.780	0.988	0.575	1
	DQ1-M3	1145	5.848	0.989	0.576	1
	DQ2-M1	1519	6.088	0.992	0.576	1
	DQ2-M2	1533	6.207	0.993	0.587	1
	DQ2-M3	1601	6.223	0.991	0.585	1
	DQ3-M1	1105	5.911	0.994	0.585	1
	DQ3-M2	1312	5.945	0.991	0.574	1
	DQ3-M3	1271	5.886	0.989	0.571	1
	DQ4-M1	1422	5.887	0.990	0.562	1
	DQ4-M2	1079	5.749	0.992	0.571	1
	DQ4-M3	1089	5.724	0.988	0.567	1
	DQ5-M1	1475	6.034	0.991	0.573	1
	DQ5-M2	1389	6.218	0.995	0.596	1
	DQ5-M3	1470	5.977	0.990	0.568	1
	DQ6-M1	1248	5.832	0.991	0.567	1
	DQ6-M2	1032	5.583	0.985	0.558	1
	DQ6-M3	1138	5.764	0.991	0.568	1
	DQ7-M1	1362	5.850	0.989	0.562	1
	DQ7-M2	1052	5.617	0.984	0.560	1
	DQ7-M3	1598	6.189	0.991	0.582	1
	DQ8-M1	1475	5.998	0.993	0.570	1
	DQ8-M2	1339	5.882	0.990	0.566	1
	DQ8-M3	984	5.734	0.991	0.577	1
	DQ9-M1	1468	6.071	0.992	0.577	1
	DQ9-M2	1253	5.766	0.988	0.560	1
	DQ9-M3	1126	5.721	0.987	0.564	1
Summer	DQ1-M1	1843	6.399	0.995	0.590	1
	DQ1-M2	1327	5.837	0.991	0.563	1
	DQ1-M3	1539	6.057	0.992	0.572	1
	DQ2-M1	1648	6.150	0.991	0.575	1
	DQ2-M2	1944	6.154	0.989	0.563	1
	DQ2-M3	1929	6.083	0.990	0.557	1
	DQ3-M1	1990	6.201	0.991	0.566	1

	DQ3-M2	1864	6.213	0.990	0.572	1
	DQ4-M1	2451	6.380	0.992	0.567	1
	DQ4-M2	2170	6.271	0.992	0.566	1
	DQ4-M3	1987	6.145	0.991	0.561	1
	DQ5-M1	2042	6.178	0.989	0.562	1
	DQ5-M2	1819	6.185	0.991	0.571	1
	DQ5-M3	1756	5.892	0.988	0.547	1
	DQ6-M1	2032	6.147	0.991	0.559	1
	DQ6-M2	1716	5.892	0.988	0.548	1
	DQ6-M3	1888	6.165	0.992	0.567	1
	DQ7-M1	1443	5.923	0.990	0.564	1
	DQ7-M2	1858	6.118	0.991	0.563	1
	DQ7-M3	1553	6.005	0.988	0.567	1
	DQ8-M1	2121	6.166	0.989	0.558	1
	DQ8-M2	2001	6.260	0.990	0.571	1
	DQ8-M3	1949	6.229	0.991	0.570	1
	DQ9-M1	2131	6.219	0.991	0.562	1
	DQ9-M2	1901	5.926	0.986	0.544	1
	DQ9-M3	1610	5.922	0.987	0.556	1
Autumn	DQ1-M1	1103	5.088	0.968	0.503	1
	DQ1-M2	1619	6.090	0.990	0.571	1
	DQ1-M3	1131	5.182	0.981	0.511	1
	DQ2-M1	1450	5.859	0.988	0.558	1
	DQ2-M2	1536	5.775	0.986	0.546	1
	DQ2-M3	1463	5.770	0.987	0.549	1
	DQ3-M1	1166	5.141	0.975	0.505	1
	DQ3-M2	1188	5.571	0.984	0.545	1
	DQ3-M3	856	4.755	0.957	0.488	1
	DQ4-M1	1073	5.437	0.985	0.540	1
	DQ4-M2	1233	5.481	0.986	0.534	1
	DQ4-M3	1371	5.724	0.987	0.549	1
	DQ5-M1	1290	5.654	0.987	0.547	1
	DQ5-M2	1381	5.780	0.990	0.554	1
	DQ5-M3	1428	5.835	0.990	0.557	1
	DQ6-M1	1592	5.849	0.988	0.550	1
	DQ6-M2	1327	5.724	0.986	0.552	1
	DQ6-M3	929	5.411	0.982	0.549	1
	DQ7-M1	1352	5.952	0.992	0.572	1
	DQ7-M2	1847	6.175	0.991	0.569	1
	DQ7-M3	1532	6.020	0.991	0.569	1
	DQ8-M1	1443	5.916	0.989	0.564	1
	DQ8-M2	1189	5.663	0.987	0.554	1
	DQ8-M3	1588	5.976	0.991	0.562	1
	DQ9-M1	1577	5.960	0.987	0.561	1
	DQ9-M2	1666	6.094	0.990	0.569	1
	DQ9-M3	1619	6.020	0.988	0.565	1

Table S2. The influence of environmental factors on bacterial communities and the significance assessment in RDA analysis

Factors	RDA1	RDA2	R^2	*P* value
Temp	0.389	-0.824	0.694	0.001
SAL	0.614	-0.028	0.315	0.001
pH	0.150	0.229	0.063	0.024
TC	-0.105	0.435	0.167	0.001
TC/TN	-0.215	0.422	0.188	0.001
SSC	-0.795	-0.315	0.609	0.001
Silt	0.252	0.161	0.075	0.006
Sand	0.315	0.125	0.095	0.008

Table S3. Sedimentary environmental parameters of different seasons at each sampling site in this study

Date	Sample	Longitude (E)	Latitude (N)	Temperature (°C)	Salinity (ppt)	pH	Clay (%)	Silt (%)	Sand (%)	TC (mg/g)	TN (mg/g)	TC/TN
2021.12	DQ1	120°30′50.16"	27°35′20.45"	14.60	6.07	8.05	29.09	67.53	3.38	11.86	0.90	13.19
2021.12	DQ2	120°31′42.68"	27°35′10.85"	14.53	5.94	7.65	22.65	74.99	2.37	10.72	0.71	15.12
2021.12	DQ3	120°32′38.46"	27°35′41.65"	14.57	13.54	8.23	32.02	65.80	2.18	13.33	1.08	12.38
2021.12	DQ4	120°34′40.82"	27°34′55.69"	13.60	20.09	8.16	30.71	68.35	0.93	12.37	0.83	15.07
2021.12	DQ5	120°34′56.44"	27°35′09.13"	13.93	18.80	7.27	28.47	69.33	2.20	12.71	1.09	11.93
2021.12	DQ6	120°35′18.17"	27°35′25.32"	14.17	18.97	8.14	24.60	74.90	0.50	12.54	0.88	14.19
2021.12	DQ7	120°36′41.72"	27°34′33.21"	15.60	17.90	7.82	32.63	65.43	1.93	12.37	0.88	14.11
2021.12	DQ8	120°36′58.53"	27°34′17.17"	13.70	20.37	8.16	28.15	71.32	0.52	13.71	1.02	13.46
2021.12	DQ9	120°38′40.54"	27°32′43.81"	14.80	15.50	7.83	31.63	64.43	3.94	13.08	0.94	13.93
2022.05	DQ1	120°30′48.69"	27°35′19.88"	21.43	4.18	7.05	36.36	63.58	0.05	12.39	1.10	11.26
2022.05	DQ2	120°31′47.31"	27°35′10.91"	20.83	1.68	7.67	26.35	73.57	0.07	10.25	0.71	14.72
2022.05	DQ3	120°32′38.03"	27°35′39.93"	21.57	6.47	7.38	40.76	59.19	0.05	12.88	1.16	11.15
2022.05	DQ4	120°34′40.88"	27°34′56.84"	22.07	13.72	7.65	32.98	65.84	1.18	12.22	0.97	12.68
2022.05	DQ5	120°34′54.95"	27°35′07.38"	21.43	12.60	8.50	30.36	67.86	1.77	12.32	0.94	13.09
2022.05	DQ6	120°35′53.78"	27°34′59.44"	21.67	13.71	7.65	36.52	63.42	0.05	11.90	0.89	13.45
2022.05	DQ7	120°36′40.67"	58°34′36.00"	24.37	18.56	7.30	41.52	58.43	0.04	12.39	0.94	13.16
2022.05	DQ8	120°36′59.41"	27°34′15.98"	21.70	12.04	6.97	38.14	61.80	0.05	12.38	0.95	13.17
2022.05	DQ9	120°38′41.03"	27°32′45.06"	22.70	14.23	7.69	41.35	58.59	0.05	13.13	1.06	12.42
2022.08	DQ1	120°30′50.55"	27°35′21.31"	29.53	8.80	7.44	30.43	69.07	0.49	11.14	0.94	11.91
2022.08	DQ2	120°31′43.40"	27°35′10.68"	31.37	25.27	7.75	28.12	69.53	2.34	11.01	0.95	11.80
2022.08	DQ3	120°32′39.17"	27°35′43.72"	30.35	18.00	7.28	20.93	76.46	2.61	12.10	1.07	11.39
2022.08	DQ4	120°34′40.45"	27°34′58.22"	28.93	26.00	7.41	29.12	66.84	4.03	12.76	1.12	11.42
2022.08	DQ5	120°34′59.56"	27°35′12.62"	29.37	32.10	8.02	34.03	64.02	1.95	11.50	0.95	12.09
2022.08	DQ6	120°35′24.55"	27°35′22.55"	29.73	24.13	7.41	31.94	66.45	1.61	11.49	0.95	12.08
2022.08	DQ7	120°36′43.06"	27°34′33.20"	30.77	29.77	7.44	32.30	66.17	1.53	11.27	0.91	12.51
2022.08	DQ8	120°36′56.26"	27°34′19.13"	28.93	25.10	7.39	30.52	68.91	0.56	13.43	0.99	13.60
2022.08	DQ9	120°38′39.92"	27°32′43.57"	31.20	32.57	7.57	32.54	63.44	4.02	13.03	1.13	11.53
2022.10	DQ1	120°30′56.72"	27°35′23.94"	21.50	28.40	7.38	33.31	66.69	0.00	11.55	1.03	11.26

2022.10	DQ2	120°31′50.94"	27°35′11.27"	20.70	25.20	7.75	31.38	63.16	5.45	11.04	0.88	12.51
2022.10	DQ3	120°32′37.38"	27°35′41.30"	20.30	30.30	7.53	34.55	60.79	4.65	11.92	1.05	11.36
2022.10	DQ4	120°33′12.03"	27°35′10.45"	21.00	28.40	7.52	38.95	61.05	0.00	11.62	0.99	11.69
2022.10	DQ5	120°34′36.07"	27°34′52.05"	21.30	28.60	7.20	37.98	62.02	0.00	11.36	0.93	12.20
2022.10	DQ6	120°34′56.61"	27°35′09.39"	20.20	33.80	8.09	36.64	63.36	0.00	12.67	0.99	12.76
2022.10	DQ7	120°36′44.03"	27°34′32.92"	21.40	32.50	7.63	36.97	63.03	0.00	12.37	1.10	11.26
2022.10	DQ8	120°37′30.64"	27°34′00.78"	22.50	27.50	7.79	28.43	70.41	1.15	10.86	0.68	16.04
2022.10	DQ9	120°38′39.73"	27°32′45.46"	21.50	26.40	8.02	37.52	62.48	0.00	12.15	1.05	11.60
